# Scaling in Free-Swimming Fish and Implications for Measuring Size-at-Time in the Wild

**DOI:** 10.1371/journal.pone.0144875

**Published:** 2015-12-16

**Authors:** Franziska Broell, Christopher T. Taggart

**Affiliations:** Department of Oceanography, Dalhousie University, 1355 Oxford Street, Halifax B3H 4R2, Canada; University of Zurich, SWITZERLAND

## Abstract

This study was motivated by the need to measure size-at-age, and thus growth rate, in fish in the wild. We postulated that this could be achieved using accelerometer tags based first on early isometric scaling models that hypothesize that similar animals should move at the same speed with a stroke frequency that scales with length^-1^, and second on observations that the speed of primarily air-breathing free-swimming animals, presumably swimming ‘efficiently’, is independent of size, confirming that stroke frequency scales as length^-1^. However, such scaling relations between size and swimming parameters for fish remain mostly theoretical. Based on free-swimming saithe and sturgeon tagged with accelerometers, we introduce a species-specific scaling relationship between dominant tail beat frequency (*TBF*) and fork length. Dominant *TBF* was proportional to length^-1^ (r^2^ = 0.73, n = 40), and estimated swimming speed within species was independent of length. Similar scaling relations accrued in relation to body mass^-0.29^. We demonstrate that the dominant *TBF* can be used to estimate size-at-time and that accelerometer tags with onboard processing may be able to provide size-at-time estimates among free-swimming fish and thus the estimation of growth rate (change in size-at-time) in the wild.

## Introduction

In 2007, Neuheimer and Taggart [1] postulated that it might be possible to collect length-at-age time series (and thus growth rate) among fishes in the wild by using archival accelerometer tags. The underlying principles for such a postulate can be found in A.V. Hill’s (1950) isometric scaling model [2] that predicts geometrically similar animals should move their limbs at a similar velocity and run or swim at the same velocity with a stride frequency that is proportional to mass^-1/3^ or length^-1^. This scaling model relies on basic physics where the work produced by a muscle during locomotion is a function of its mass and thus the resultant kinetic energy will depend on the mass and the velocity squared. Consistent with this model, observations from a range of free-swimming seabirds and mammals, presumed to be swimming ‘efficiently’ [3], suggested that the animals adopted cruising speeds that are independent of body size and that the associated dominant stroke cycle frequencies scaled with mass^-0.29^ [3]. For geometrically similar soaring seabirds [4] and penguins [5], the dominant stroke cycle frequency was shown to be proportional to mass^-0.30^ and mass^-0.28^ respectively, and in each the scaling exponent was not significantly different from -1/3 [3,4,5]. Most recently, Gazzola et al. [6] proposed that for turbulent flow regime, at a given speed *u*, tail beat frequency is inversely proportional to tail beat amplitude. Given the experiments by Bainbridge [7] that indicate tail beat amplitude is proportional to length at any given speed, tail beat frequency therefore is inversely proportional to length, which provides a new theoretical justification for such observations [3,4,5]. Such scaling relationships are expected to hold for large (adult) swimmers in the inertial flow regime or as long as swimmers are not trading efficiency for another performance parameter such as speed, with a likely nonlinear relationship in laminar and intermediate flow regimes (e.g.,[8]).

Notably, the above multi-species studies [3] included only two species of fish and each with a small sample size; Japanese flounder (*Paralichthys olivaceus*), *n* = 5, and chum salmon (*Oncorhynchus keta*), *n* = 2. Not only does the limited sample size not allow us to firmly conclude that the scaling law does apply for fish species, for the flounder, the dominant stroke frequency (tail beat frequency, *TBF*) was anomalously low relative to the fitted inter-specific scaling model. This was attributed to the estimates being derived from potentially ‘inefficient’ swimming [3], and thus contradicts the assumption of ‘efficient’ locomotion [2,3,5], although there is no clear definition of efficient swimming for fishes.

Efficiency can be defined at several organizational levels such as mechanical efficiency (propeller efficiency) or metabolic efficiency (entire organism). In swimming and locomotion research, studies define efficiency as the ratio of useful to total work or power. To assess propulsive performance, studies often calculate hydrodynamic or mechanic efficiency as the ratio of useful over total work done by the propeller (e.g., [9, 10]); swimming or metabolic efficiency include muscle and respiratory processes to calculate efficiency (e.g., [11]) or measure oxygen consumption (energy expenditure) in closed respirometer experiments during steady-swimming. Efficient swimming is also assumed to occur during high-energy-cost movements, e.g., during migration or feeding bouts [3]. Efficiency during steady-swimming has further been measured using the Strouhal number, which relates tail beat amplitude and frequency to swimming speed [3,6,12]. Some of these efficiencies are only applicable in a narrow range of behaviours, e.g., steady-swimming. According to Sato et al [3], unlike breath-holding mammals, reptiles and birds, fish do not necessarily swim efficiently, at least at top speed, and thus Hill’s isometric scaling may not hold. Furthermore, the deviations apparent for the studied fish species in the interspecific scaling relation should not be unexpected since interspecific scaling relations are known to differ from intraspecific relations (e.g., [13,14]) given ontogenic constraints [13] and complications that arise from fitting a bivariate relation to a multivariate problem [15].

To our knowledge, there is a very limited literature examining the above scaling relations among sizes and/or species of (adult) fish, and this is likely due to the inherent difficulty of obtaining such data on free-swimming fish. The few studies that have been published do not include sufficient data or information to allow the conversion of measurements to a common size-related parameter. The consequence is that most analyses of the relations between fish size and locomotion remain theoretical [2,16,17,18]. However, advances in digital accelerometer tags now provide a method of obtaining the necessary swimming data in the laboratory [19,20], and in the field [21,22,23], and such data have been used to quantify behavioural states and rates and to estimate parameters such as energy expenditure and swimming activity [21,22] through the extraction of tail beat frequency estimates [3,21,22,23].

Quantifying relationships between size and movement may help reconcile co-evolutionary mechanisms [4] and help address the ecological implications of size-dependent locomotion [24]. It will also have practical applications in fisheries science because fish size influences metabolic rate, physiology, and ingestion rate, and thus growth, maturity and fecundity and ultimately abundance [24]. Size-at-age measures are also essential in fisheries science because virtually all population and assessment models involve some component of growth-rate-dependent demography that varies among cohorts and age-classes. Measuring size-at-time and inferring growth rate in wild fish is inherently difficult, and to date can only be achieved over relatively long time scales using mark and recapture techniques or by using post-mortem morphometrics such as otolith microstructure that have their own inherent uncertainties [25,26].

Here, we suggest a new method of measuring size-at-time in fish, and potentially growth rate, based on Hill’s isometric scaling. We hypothesized that if it is possible to establish a within- or among-species allometric relationship (model) that relates fish size to tail beat frequency from acceleration data, then such a model could be used to estimate size-at-time, and thus growth rate over time in the wild. We therefore collected acceleration data and the derived tail beat frequency estimates among a size range of free-swimming saithe (*Pollachius virens*), a species widely studied in kinematic experiments [27,28,29] and analytical models [30,31], and shortnose sturgeon (*Acipenser brevirostrum*) [32,33,34].

## Materials

### (a) Study animals

Saithe (*n* = 18) of fork length (*l*; m) ranging from 0.26 to 0.56 m (average ± SD, 0.41 ± 0.089 m) with mass (*m;* kg) between 0.18 and 1.6 kg (0.93 ± 0.48 kg) were collected near Nova Scotia, Canada. Accelerometry data were obtained from the fish swimming freely at Dalhousie University in a large mesocosm with a diameter of 15.24 m, a depth of 3.54 m at the perimeter and 3.91 m at the centre, and a volume of 684 m^3^ natural seawater held at 9°C ± 2. Swim trials were conducted over 9 trial-days spanning a month. Each individual fish swim trial lasted between 24 and 29 h with a recovery period of two to five days.

Shortnose sturgeon (*n* = 22) with *l* ranging from 0.56 to 1.2 m (0.79 ± 0.18 m) were used for free-swimming trials. Individual mass (not measured) was estimated using a mass-at-length relationship for adult fish (Fig 7 within [35]) based on average age (collected in 1998–1999 in the Saint John River, NB, Canada and held in captivity at the nearby Mactaquac Biodiversity Facility). Accelerometry data were collected over a one-week period in two 11 x 11 m-wide, 1 m-depth, outdoor flow-through tanks held at an ambient river-water temperature of 15.5 ± 0.5°C.

### (b) Accelerometers

We used three tri-axial accelerometer tag models (Maritime bioLoggers, Halifax, Canada). For saithe, we recorded tri-axial acceleration at 50 Hz (10-bit resolution) at ± 4 *g*_*o*_. Saithe exceeding 40 cm were tagged with the MBL PT-1 (50 mm length, 23 mm diameter, 18.8 g in air). Smaller fish were tagged with the MBL PT-2 (25 mm length, 17 mm width, 11 mm height, 6.1 g in air). Shortnose sturgeon were tagged with the MBL PT-0 (53 mm length, 35 mm width, 15 mm height, 14.6 g in air) sampling at 550 Hz (10-bit resolution) at ± 3 *g*_*o*_.

### (c) Saithe swim trials

Saithe were anaesthetized with MS222 (40 mg l^-1^), measured for *l* and *m* and tagged using Petersen Disc tags (see [19] for tag attachment details) and before each swim trial an accelerometer was attached (in a removable manner) to the disc. Fish swam *ad libitum* for 48 hours with no external stimulus save a natural daylight cycle. Following each trial the accelerometer was detached and the animals recovered in a holding tank (2 x 2 m). At least 4 h of free-swimming accelerometer data were collected for each individual fish for a total of 845 h of data.

### (d) Shortnose sturgeon swim trials

Sturgeon were measured for *l* and tagged using a spandex belt (housing the accelerometer) wrapped around the caudal peduncle, anterior to the dorsal fin. Fish were randomly assigned to the swim-trial tank (isolated) or the holding tank (communal) where they were allowed to swim *ad libitum* in a continuous but spatially variable current (0.0 to 0.3 ms^-1^) in natural daylight conditions. At least 0.5 h of free-swimming data were collected for each individual for a total of 18 h of data.

## Methods

### (a) Estimating dominant tail beat frequency (*TBF*) from acceleration

Tail beat is a non-stationary periodic oscillation in the acceleration time series [3,21,22]. Thus, to extract continuous, steady swimming data from accelerometer records, we defined the steady swimming segment such that *TBF* (Hz) did not statistically vary within a segment. We then developed a *TBF* extraction algorithm that was based on zero-crossings (S1 Fig; see also [36,37]) with adaptive window lengths. The algorithm was applied to a time series after removing the high-frequency noise (IIR Butterworth filter with a 15 Hz cut-off). An initial window length was chosen to resolve the average, expected, species-specific *TBF* [27,34], e.g., 2 seconds for saithe. Steady swimming segments were those where the period between zero-crossings was ‘stable’; established by comparing the variability in the zero-crossing intervals (i.e., beat periods, *Δt*’s) to a stability threshold that was based on the range of *Δt*’s in the entire series (*Δt*_*max*_ *−Δt*_*min*_) multiplied by a scaling parameter. Segment length was then established by statistically comparing consecutive windows of *TBF* estimates based on nonparametric mean comparisons. Each series of consecutive windows of relatively invariant *TBF* was assumed to represent a steady swimming segment. To estimate the dominant *TBF* we combined segments from the same individual among multiple swim trials. The algorithm above was used to extract a list of frequencies and corresponding segment lengths. Stable *TBF* segments within the upper 10^th^ percentile, by duration, were used for analyses, again assuming they represented steady swimming. These segments were then used to establish weighted histograms, means, medians and standard deviations, where the weights corresponded to the length of each segment with a stable *TBF*.

### (b) Species-specific analyses

We calculated weighted log-log regressions for each species using the moments of the *TBF* distribution. The response variable was log_e_ of the median *TBF* obtained from the weighted *TBF* distributions for each individual, and the predictor variable was log_e_ of *l* or *m*. The regression weights were determined using the variance of the log_e_ median *TBF* [38, 39].

### (c) Average swimming speed

**Fig 1:**
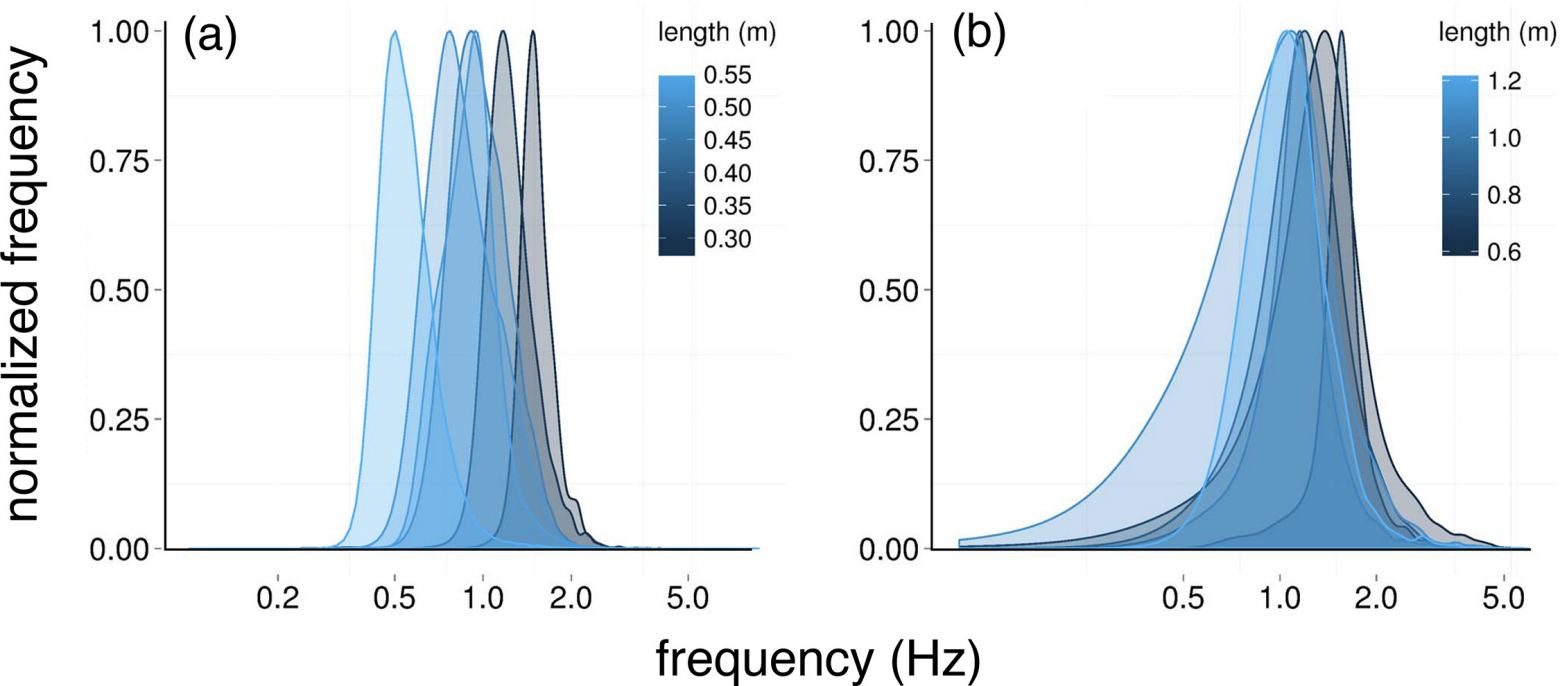
Examples of normalized tail beat frequency (*TBF*, Hz) density distributions from accelerometer records of free-swimming (a) saithe (*n* = 6) and (b) sturgeon (*n* = 6) based on weighted histograms of *TBF* extracted using the zero-crossing algorithm.

Absolute average swimming speed, *u* (ms^-1^), was estimated as a function of dominant *TBF* and *l* based on literature models (see S1 File). For saithe we used the empirical relation provided by Videler and Hess (Table 1 within [27]) where *u* = *l* • 0.977 *TBF* ^0.883^. For shortnose sturgeon we used the relation from Long (Fig 1 within [34]) where *u* = *l* • (0.005 + 0.138 *TBF*).

Algorithm computations and statistical analyses were performed using R [40], and MATLAB 8.0 [41]. Unless otherwise noted, all estimates are provided as the average estimate plus or minus one standard deviation. Subscripts indicate species (*P*, saithe and *S*, sturgeon).

Ethics. Fish care and protocols for fish holding, surgery, tagging, and swim trials were approved by Dalhousie University (saithe, Permit 12–049) and Mount Allison University (sturgeon, Permit 10–16) in accordance with the Canadian Council for Animal Care standards.

## Results

### (a) *TBF* distributions

*TBF* estimates for saithe were log-normally distributed (Fig 1A) with medians ranging from 0.6 to 2 Hz (1 ± 0.3 Hz) across all sizes. Estimates for sturgeon were near log-normal (Fig 1B) with medians ranging from 1.1 to 2.4 Hz (1.5 ± 0.3 Hz). For each species log_e_ *l* was normally distributed (Anderson Darling: saithe *p* > 0.01; sturgeon *p* > 0.05).

### (b) *TBF* as a function of length

In general, fish length and mass tend to be strongly correlated (for saithe from our data, *r* ^*2*^ = 0.93 and for sturgeon from Dadswell [35], *r* ^*2*^ = 0.99). To avoid redundancy we focus here on length and the allometric relationships are summarized in Tables 1 and 2. Equivalent results based on mass are provided in S1 File.

**Table 1:** Summary of allometric relations among swimming parameters based on dominant tail beat frequency (*TBF*, Hz), estimated swimming speed (*u*, ms^-1^), and fork length (*l*, m) in two fish species. Subscript, *sd*, indicates standardized by the species-specific average, the *p*-value indicates significance between predicted and observed *β* where the 95% confidence interval (CI), coefficient of determination (*r* ^2^) and sample size (*n*) are provided.

Species	Relation	Exponent (*β*)*	95% CI for *β*	Predicted *β* ^a^	*p*	*r* ^2^	*n*
*P*. *virens*	*TBF* ∝ *l* ^*β*^	-0.99 (±0.15)	[-1.3; -0.68]	-1	0.87	0.73	18
	*u* ^†^ ∝ *l* ^*β*^	0.12 (±0.13)	[-0.16; 0.40]	0	0.30	0.01	18
*A*. *brevirostrum*	*TBF* ∝ *l* ^*β*^	-0.89 (±0.094)	[-1.09; -0.69]	-1	0.29	0.82	22
	*u* ^‡^ ∝ *l* ^*β*^	0.12 (±0.092)	[-0.067; 0.32]	0	0.19	0.01	22
Combined	*TBF*_sd_ ∝ *l* ^*β*^_sd_	-1.0 (±0.097)	[-1.2; -0.80]	-1	0.80	0.73	40
	*u*_sd_ ^†^ ^‡^ ∝ *l* ^*β*^_sd_	0.12 (±0.086)	[-0.054; 0.29]	0	0.17	0.05	40

^a^Predicted value based on [1]

*from log-log ordinary least square slope

^†^using *u* and *TBF* model from [27]

^‡^ using *u* and *TBF* model from [34]

**Table 2:** Summary of regression models for predicting fork length (*l*, m) as a function of dominant tail beat frequency (*TBF*, Hz) for saithe (*P*. *virens*) and sturgeon (*A*. *brevirostrum*) where the proportionality constant with the 95% confidence interval (CI), exponent (*β*) with standard error (SE) and 95% CI, coefficient of determination (*r* ^2^) and sample size (*n*) are provided.

Species	Relation	Proportionality constant, *b* * [95% CI]	Exponent, *β* *(±SE) [95% CI]	*r* ^2^	*n*
*P*. *virens*	*l* ∝ *b TBF* ^*β*^	0.47 [0.39; 0.45]	-0.74 (±0.11) [-0.97; -0.50]	0.73	18
*A*. *brevirostrum*	*l* ∝ *b TBF* ^*β*^	1.1 [0.97; 1.1]	-0.91 (±0.10) [-1.1; -0.70]	0.81	22

* from log-log ordinary least square intercept and slope

**Fig 2:**
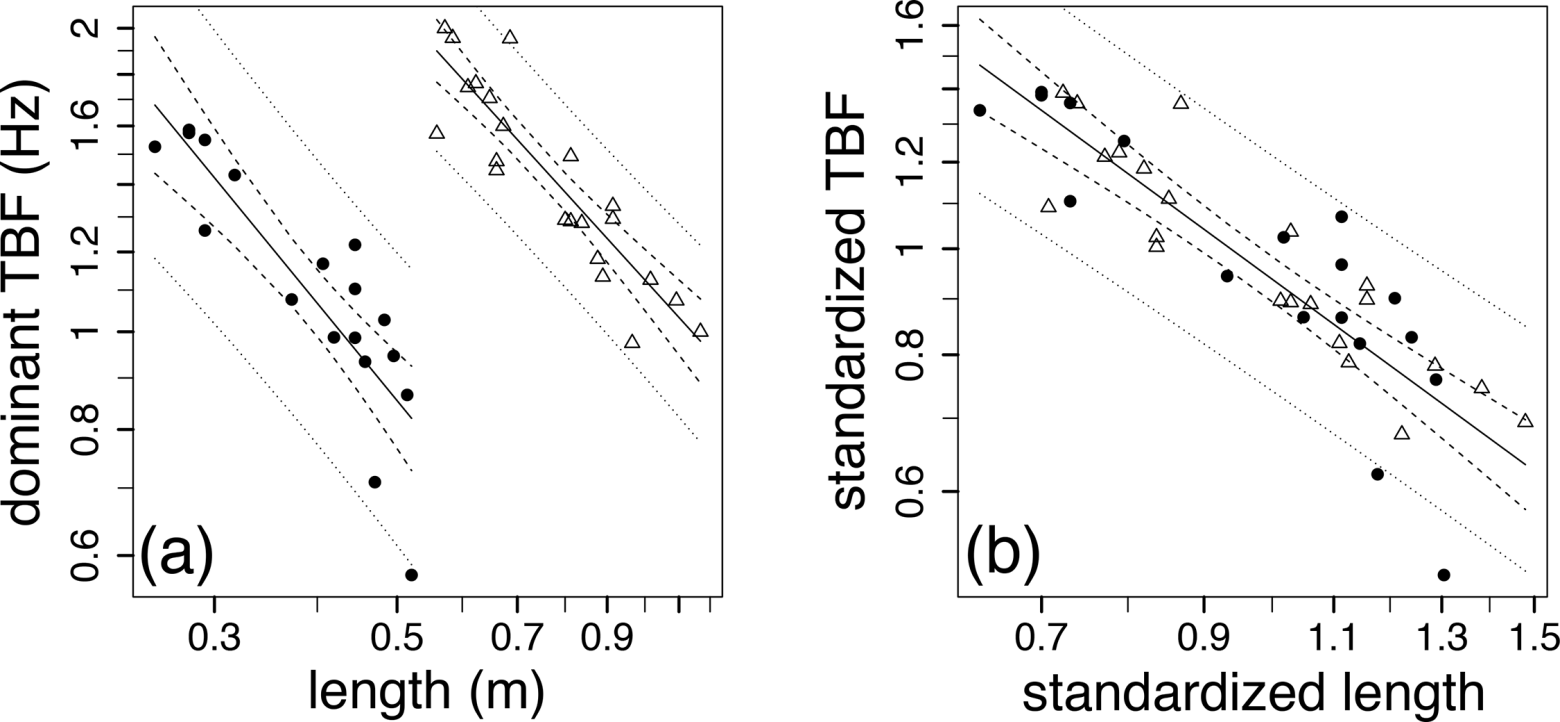
Log-log relations between (a) dominant tail beat frequency (*TBF*, Hz) and length (m) and (b) standardized *TBF* in relation to standardized length for saithe (solid circles, *n* = 18) and sturgeon (open triangles, *n* = 22), where the weighted ordinary least square regressions (solid line) are bracketed by the 95% confidence intervals (CIs) around the regression (dashed line) and the unweighted 95% CIs around the predictions (dotted lines).

As shown in Fig 2A and Table 1, the dominant (median) *TBF* was a strong function of *l* for each species: for saithe

$${TBF}_{P}\;=\;0.43\;l^{-0.99}\;(n=18,\;r^{2}\;0.73),$$ (1)

and for sturgeon

$${TBF}_{S}\;=\;1.1\;l^{-0.89}\;(n=22,\;r^{2}=0.82).$$ (2)

The above length exponents were not different (*p* = 0.64) between species and the 95% confidence intervals (CIs) for the slopes each bracketed a slope of -1.0 (Table 1) as predicted [2,3,4,5].

The species-specific relations could not be combined for phylogenetic analyses [3,4,5] because average *TBF* and length among the sturgeon were each greater than among the saithe (Student’s t-test, *p* < 0.05) and thus the difference between their respective proportionality constants. When *TBF* estimates and lengths were scaled by the species-specific average *TBF* and average length, the *TBF* for the combined species was again a strong function of *l* (Table 1, Fig 2B);

$$TBF\;=\;0.94\;l^{-1.0}\;(n=40,\;r^{2}=0.73).$$ (3)

### (c) Swimming speed as a function of length

**Fig 3:**
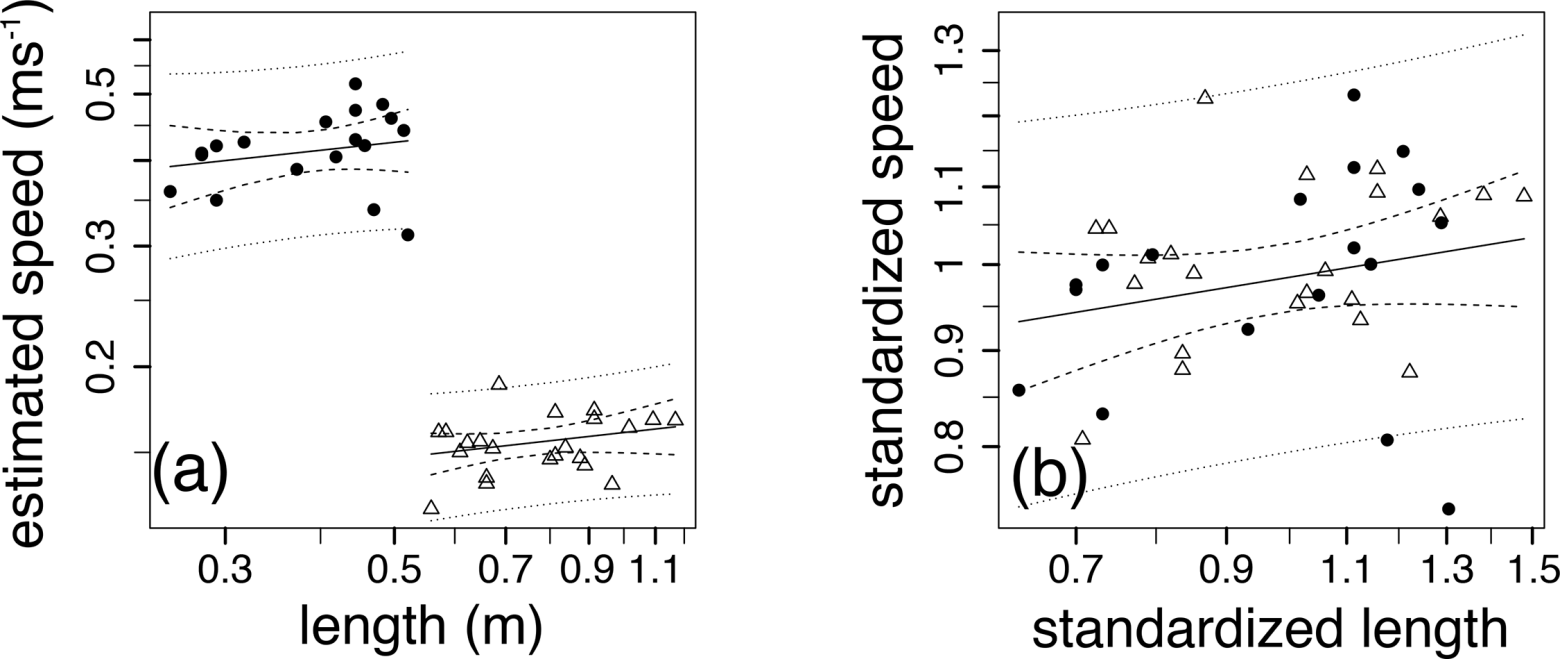
Log-log relations between (a) swimming speed and length and (b) standardized swimming speed and standardized length for saithe (solid circles, *n* = 18) and sturgeon (open triangles, *n* = 22) where weighted ordinary least square regressions (solid line) are bracketed by the 95% confidence intervals (CI) around the regression (dashed lines) and unweighted 95% CI around the predictions (dotted lines).

The derived absolute swimming speed estimates, which were estimated from the literature [32,34] were normally distributed (Anderson Darling, *p* > 0.5, Fig 3) with average speeds of 0.41 ± 0.05 ms^-1^ for the saithe, and 0.15 ± 0.01 ms^-1^ for sturgeon. The response variable, *l*, was log transformed to stabilize the variance. Within species, average swimming speed was independent of *l* (*p* > 0.01, Table 1, Fig 3A). While the length exponents for each species were not different (Table 1.), the proportionality constants were (Fig 3A), again preventing inter-species comparison. When standardising the response and predictor variables by the species-specific averages, the standardized average swimming estimates were independent of *l* (weighted ordinary least squares, *p* = 0.17, Table 1, Fig 3B).

### (d) Length as a function of *TBF*

**Fig 4:**
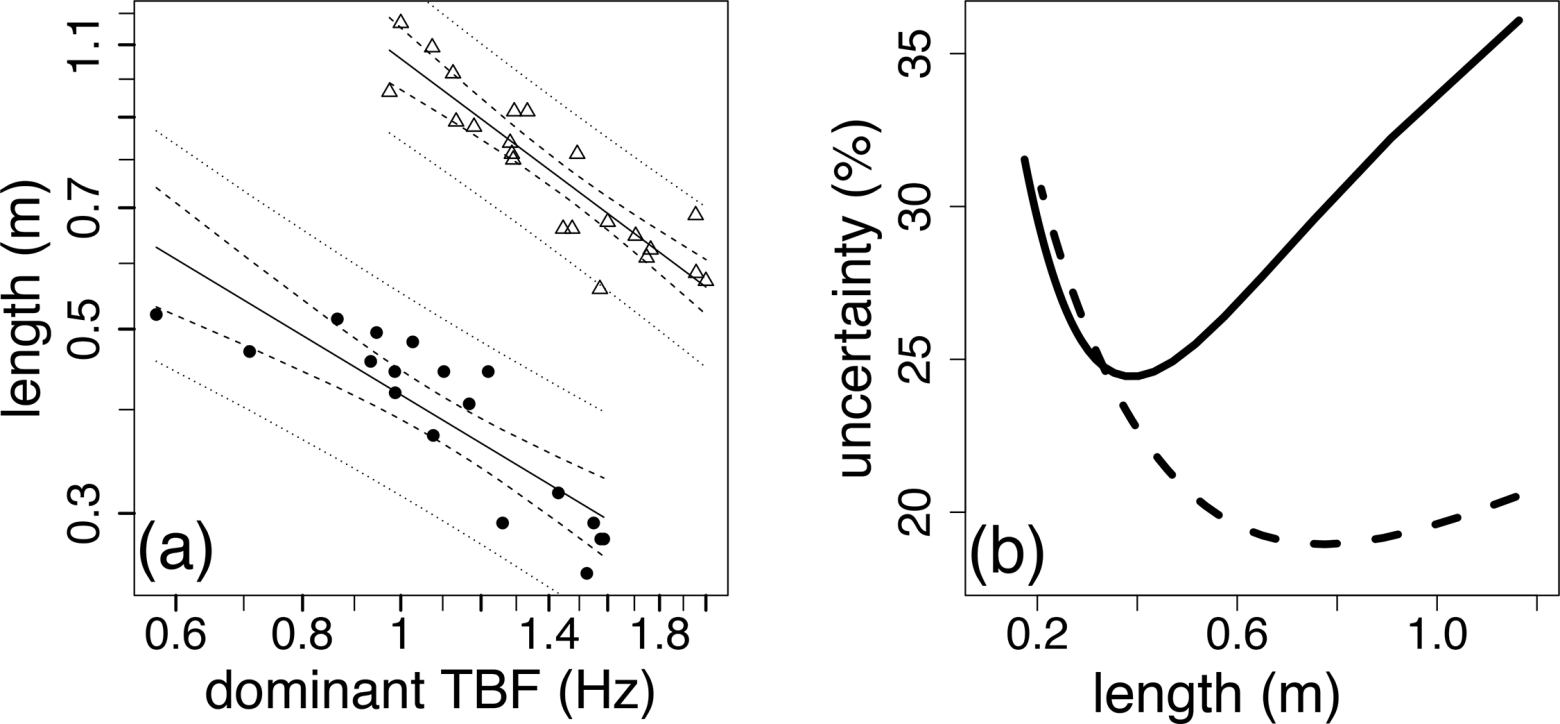
(a) Log-log relations between dominant tail beat frequency (*TBF*, Hz) as predictor and length (m) for saithe (solid circles, *n* = 18) and sturgeon (open triangles, *n* = 22) showing ordinary least square regressions (solid line) bracketed by the 95% confidence intervals around the regression (dashed lines) and predictions (dotted lines), and (b) prediction uncertainties, *P*_*U*_ as a function of length (m) for saithe (sold line) and sturgeon (dashed line) expressed as *P*_*U*_ = 100 *t*_*0*.*975*,*n-2*_ *SE*_*lp*_/*l*_*p*_ where *lp* is the model prediction and *SE*_*lp*_ is the associated standard error for the prediction.

Given Eq 1 and Eq 2 above, it is not surprising that from a prediction perspective length was a function of dominant *TBF* (Fig 4A, Table 2) where *l* = 0.47 *TBF* ^− 0.74^ (*r* ^2^ = 0.73), and also for sturgeon (Fig 4A, Table 2) where *l* = 1.1 *TBF* ^− 0.91^ (*r* ^2^ = 0.81). The species-specific exponents were different (*p* = 0.003) and the exponent for sturgeon as not different from -1 (*p* = 0.4), and for saithe it was marginally different from -1 (*p* = 0.03). Fig 4B illustrates the uncertainty in size predictions for each species based on the maximum sizes (~1.2 m) typically observed in nature [35,42]. For each species, the 95% prediction uncertainty was expressed as *P*_*U*_ *= t*_*0*.*975*,*n-2*_ *SE*_*lp*_ */l*_*p*_, where *l*_*p*_ is the model predicted size and *SE* is the associated standard error. Due to the fish lengths available for the study, the greatest confidence for prediction was at intermediate sizes (> 0.2 and < 0.6 m for saithe and > 0.4 m for sturgeon). The least uncertainty for saithe was at 0.4 m (~25%) and for the sturgeon at 0.7 m (~18%), while the largest uncertainty for saithe was at 1.2 m (~36%) and for sturgeon at 0.2 m (~30%).

When *TBF* observations collected from a comparable sturgeon species in the wild during ascent (Chinese sturgeon, *Acipenser sinensis* [43]) are used as model input (1.08 Hz, 0.77 Hz, 0.91 Hz), length predictions (1.03 m, 1.39 m, 1.19 m) are between 4–14% when compared to the measured length (0.95 m, 1.22 m, 1.15 m, respectively), which provides more confidence in model results.

## Discussion

It has been historically difficult to examine allometric scaling relationships between swimming speed, tail beat frequency and size in fish beyond the theoretical [2,16,17,18], largely due to the difficulty of obtaining data on free-swimming fish across a suitable size range [44]. Here, we quantified and validated theoretical allometric scaling relationships for two different free-swimming fish species of relatively large but different size ranges by using accelerometer tags. Using the acceleration records from the free-swimming saithe and sturgeon, we developed a signal-processing algorithm that extracts, from a non-stationary signal, the dominant tail beat frequency (*TBF*) for steady swimming and demonstrated that *TBF* is a function of size for each species; scaling with length^-1^ and mass^-0.29^. These exponents are not statistically different from Hill’s isometric prediction that *TBF* scales with length^-1^ and mass^-1/3^ [2] and results from the species-specific independence between absolute average swimming speed and each of length and mass [3]. These results subsequently allowed us to demonstrate that dominant *TBF* can be used to predict species-specific length-at-age with prediction uncertainties as low as 18%, thus providing a novel method for estimating length-at-age in the wild.

Similar to our results, Sato et al. [3] provided a unifying scaling model that predicts that similarly sized animals, among many large and widely disparate species (0.5 to 1600 kg), should display the same dominant stroke cycle frequency at a given mass (or length). In contrast, we found species-specific differences manifested as different model proportionality constants, despite the presumed geometric similarities. Such differences may be masked in Sato et al. by the large species-size range they analyzed and when their data were reanalyzed from a species-specific perspective, the differences emerged. Our results above predict that the dominant *TBF* for sturgeon is twice that of saithe at the same size (and the observed was as much as three fold higher), and we estimated that the absolute swimming speed for saithe, while necessarily taken with caution, was lower in sturgeon of the same size. Since our results also indicate that absolute swimming speed and size are independent for each species [3], this difference may be due to differences in pressure load [6]. Morphological limitations, such as high drag resulting from body form and external bony scutes, exacerbated by low thrust from a heterocercal tail [33], may further account for reduced swimming ‘efficiency’ [33] among sturgeon relative to similarly sized saithe. Differences between interspecific and intraspecific scaling are to be expected given that intraspecific scaling faces ontogenetic constraints. Interspecific scaling coefficients can also be expected to differ (e.g., scaling across mammalian leg bones versus scaling within bovine leg bones, [45]). Such complications arise from fitting a bivariate relation to a multivariate problem [15].

A theoretical basis for the observed species-specific differences may be found by extending the theory provided by Gazzola et al. [6]. At very high Reynolds numbers (*Re* > 10^3^ to 10^4^, as for all fish studied here), and balancing thrust and skin drag for elongated swimming bodies, it follows [6], that

u∝fA (4)

where *A* relates to the tail beat amplitude. For fish of a given size, when swimming at high speeds, they maintain an approximately constant length-specific tail beat amplitude [6,7] which can be defined as *A = al*, where *a* is some species-specific coefficient; e.g., *a* = 0.18 for saithe [6]. Given that *u* ∝ *f a l*, and if *f* ∝ *l* ^-1^ as indicated by our observations and those of others, then *u* must be constant. Similarly, at a given speed *u*, *f* relates to length as *f* ∝ *(al)* ^-1^ and this not only provides a mechanical justification for the observed scaling relationship, it also offers an explanation for the differences in the species-specific models; i.e., the species-specific coefficient, *a* (that scales the constant tail beat amplitude with body length), affects the constant in the scaling relationship accordingly. Until it can be demonstrated that length specific stroke amplitude (*a*) is species independent, it is difficult to validate an interspecific relationship. Not only does this advance the scaling between *TBF* and length, it also implies that for a given species, fish swimming speed is independent of length.

Hill’s isometric model [2] is assumed to hold only for efficient movement; i.e., ‘natural swimming behaviour of free-ranging animals in contexts where they are expected to swim efficiently’ [3]. While our studies did not allow the observation of movements unequivocally known to be associated with the above contexts, the predicted relationship was validated. This was achieved by estimating the dominant *TBF* from the acceleration record using a novel algorithm and discarding unsteady swimming movements. Additionally, we confirmed that for the longest 10^th^ percentile of the continuous steady swimming segments used in the analysis, swimming could be shown to be efficient by calculating the Strouhal number (*St*); a commonly used index of efficient swimming (*St = Af/u*, where *A* = tail beat amplitude, *f* = tail beat frequency and *u* = swimming speed [3,6]). For example, *St* for saithe was calculated using the estimated swimming speed [27] with a tail beat amplitude of 0.18 *l* [6]. For the stable *TBF* segments the *St* estimates were between 0.22 and 0.23; close to that expected for saithe during efficient swimming [31]. Additionally, it can be shown that dominant *TBF* is linearly proportional to maximum *TBF* (S1 File, S4 Fig, S3 Table), which further, in this context, validates its use as a proxy for efficient swimming. Our results are coherent with the Sato et al [3] cross-species model and consistent with the prediction by Hill who equated the work a muscle produced (∝ *m*) at a given frequency (*f*) with the mechanical power required to counteract drag. However, our results are in stark contrast to the theoretical suggestions presented by Bainbridge [7] that *u* ∝ *l* ^0.39^ for high Reynolds numbers, by Gray [17] that *f* ∝ *l* ^-0.44^, and by and Wu [46] that *f* ∝ *l* ^-0.88^. If swimming speed is proportional to length, with some exponent *c*, then *f* scales with *l* ^c-1^ as predicted by Webb [47,48]. For example, at maximum sustained tail beat frequency, *TBF*_*MS*_ is proportional to *l* ^-0.51^ (see S1 File). Based on Eq 4, this occurs if *u*_*MS*_ ∝ *l* ^0.49^, which is close to the predictions of Webb [24,48]. This suggests that the above theoretical models based on muscle power output are insufficient in explaining the underlying mechanism(s) for fish. The most likely reason is the discrepancy between (theoretical) swimming speeds and the swimming modes considered (e.g., critical, maximum, sustained, etc.) and how poorly those modes correspond to the observed dominant swimming mode, which may in fact be the preferred swimming mode adapted to by a given fish/species [2,3]. This may help explain why our estimates of average *TBF* are much lower than those predicted by Videler and Hess [27] and Videler and Wardle [49]; and closer to estimates made for comparable species in the wild, such as Chinese sturgeon (*Acipenser sinensis* [43]), trout (*Oncorhynchus mykiss*, [22]), and sockeye salmon (*Oncorhynchus nerka*, [50]).

### Implications for measuring size-at-age in the wild

We have demonstrated that it is possible to predict size from dominant *TBF* by using species-specific models based on accelerometer tags mounted on free-swimming saithe and shortnose sturgeon of various sizes. While the confidence intervals for each of the models are reasonable, the large prediction intervals may not yet provide a suitable alternative to the conventional methods of estimating size-at-age to infer growth rate. We think that the model coefficients of determination and the prediction intervals, and therefore length prediction certainty, should improve if such studies were repeated over longer periods within more natural environments using a greater range of lengths. Based on the theoretical prediction as outlined above, and demonstrated by empirical data for sturgeon, this scaling exponent is predicted to be -1 since *f* ∝ *(al)* ^-1^ and therefore *l* ∝ *(af)* ^-1^. In summary, for a given species, size is directly and inversely related to the dominant tail beat frequency, thus allowing the estimation of size from the dominant *TBF* in the lab or in the wild, as shown here from an empirical and theoretical perspective. Differences that fish experience in the lab vs. field environment (currents, schooling, behaviour), may certainly affect the observations and associated prediction model. Some of our observations may allow us to predict such effects on the prediction model. The experimental set-up leads us to conclude that the effect of currents is expected to be minimal, since for sturgeon, which were exposed to variable currents, the scaling relationship did not seem to be affected. This is not surprising as for most, but not all fish in the ocean, lakes and large rivers, the current eddy-field is much larger than the fish. Furthermore, when predicting length with *TBF* from wild sturgeon during ascent (*Acipenser sinensis* [43]), the high prediction accuracy (4–14%) affords some confidence in the model. Schooling may have an effect on steady swimming. For example, saithe are a schooling species and the data were collected while fish were spending some time swimming in schools and some time swimming solitary. However, these differences in swimming behaviour were not apparent in the scaling relationship. Sturgeon were randomly assigned to a swim tank where they were allowed to swim solitarily or with conspecifics. Again, when data were pooled by experiment type (solitary vs. communal) no difference appeared. We do not believe that behavioural differences in the wild will have a significant effect on the scaling relationship since, e.g., feeding behaviour and other movement-related behaviour (spawning, escape etc.) is often exhibited by non-steady and burst acceleration swimming (e.g.,[51]). Since the proposed algorithm removes such swimming bouts prior to estimating the dominant tail beat frequency, such behavioural differences should not affect the results.

For the model predictions to prove useful in measuring size-at-time (and eventually growth) a study similar to ours needs to be conducted using fish as they grow to unambiguously demonstrate that within individual variation over time is less than within and among size-class variations. Such a study would determine the utility of the model and using accelerometry to estimate size-at-time (and growth) in the wild as a reasonable alternative to conventional methods such as post-mortem morphometrics that include otolith microstructures. While it is generally accepted that otolith growth is a ‘running average’ of somatic growth [52] there are uncertainties in the accuracy of back-calculations of fish size or growth rate from otolith size due to reader bias [53,54], or bias introduced by the way the otolith is prepared [1,55].

The use of more replicates among size-classes and across a larger size-range will likely improve the prediction interval and explained variance by reducing within-class variation that is likely related to individual variability in the short-term response to tagging-induced stress. Adding additional parameters that scale with length, and (or) by combining the knowledge of the initial fish size at capture, along with the theoretical characteristics of growth potential, could further improve the model prediction of size over time by using the prediction from the scaling model in a state-space framework. The indirect observations of length from the scaling relationship provides the final element to be combined with an initial measurement of fish length (when tagged) and a prediction from fish growth theory, which may even include additional predictors (such as temperature [1]) to construct a state space model of fish length at time. Such a model may provide a more reliable time series of length-at-age (see S1 File). For example, maximum velocity (or maximum tail beat frequency) scales with length as shown in S4 Fig and S3 Table using 11 species drawn from the literature [7,48], and maximum *TBF* is proportional to *l* ^-0.51^ (*n* = 44, *r* ^2^ = 0.41). However, it is difficult to observe maximum *TBF* in nature and likely more difficult to determine when maximum *TBF* is reached. Furthermore, when such a model is used to calculate maximum *TBF* for the saithe and the sturgeon, maximum *TBF* was linearly related to dominant *TBF* for saithe with a slope of 2.6 (*n* = 18, *r* ^2^ = 0.79) and for sturgeon with a slope of 1.5 (*n* = 22, *r* ^2^ = 0.78). Therefore, adding this parameter to the scaling model would prove redundant.

Assuming our prediction models can be further validated in nature, and that micro-processing technology of archival accelerometer sensors can employ an *a priori* determined algorithm that continuously (or duty-cycled) calculates size-at-time, then the *in situ* estimation of size-at-time and growth rate could be achieved. The algorithm that relates dominant *TBF* to size has the potential of providing a powerful tool in estimating size-at-time in the wild; something yet to be achieved. Since this algorithm is based on sampling a known log-normal *TBF* distribution, which would require ~30 measurements for reliable estimation (Central Limit Theorem), and the dominant *TBF*s among comparable species can be sampled at a low frequency (~ 15 Hz), then the accelerometer-tag power consumption would be comparably low.

## Supporting Information

S1 Fig Tail beat frequency extraction algorithm flow chart. Flow chart of the zero-crossing algorithm used to extract time-varying tail beat frequency (*TBF*) where *Δt* is the beat half period, σ is the standard deviation, *E(Δt)*_*i*_ is the average beat half period within window *i* and ThS* is a tuning parameter that is a function of the range of all periods (i.e., *Δt*_*max*_ *—Δt*_*min*_) in the time series multiplied by a scaling value. Finding zero-crossings is based on [11]. A typical species-specific initial window length, *W* for e.g., saithe is 2 seconds.
(PDF)

S2 Fig Relationship between dominant tail beat frequency and mass. Log-log relations between (a) dominant tail beat frequency (*TBF*, Hz) and mass (kg) and (b) standardized *TBF* in relation to standardized mass for saithe (solid circles, *n* = 18) and sturgeon (open triangles, *n* = 22). Weighted ordinary least square regressions (solid line) are bracketed by the 95% confidence intervals (CIs) around the regression (dashed line) and the unweighted 95% CIs around the predictions (dotted lines).
(EPS)

S3 Fig Relationship between swimming speed and mass. Log-log relations between (a) swimming speed (ms^-1^) and mass (kg) and (b) standardized swimming speed and standardized mass for saithe (solid circles, *n* = 18) and sturgeon (open triangles, *n* = 22) where weighted ordinary least square regressions (solid line) are bracketed by the 95% confidence intervals (CI) around the regression (dashed lines) and unweighted 95% CI around the predictions (dotted lines).
(EPS)

S4 Fig Maximum Tail Beat Frequency as a function of length and dominant Tail Beat Frequency. (a) Log-log relation between maximum tail beat frequency (*TBF*) in relation to length based on 11 species (*n* = 44) from [7,48] and (b) linear relation between maximum tail beat frequency (*TBF*) and dominant *TBF* for saithe (filled circles, *n* = 18) and sturgeon (open triangles, *n* = 22) with ordinary least square regressions (solid line) bracketed by 95% confidence intervals around the regression (dashed line) and the predictions (dotted lines).
(EPS)

S1 File Scaling mass and tail beat frequency, and details on the calculation of swimming speed. (DOCX)

S1 Table Allometric relations among swimming parameters and body mass. Summary of allometric relations among swimming parameters based on dominant tail beat frequency (*TBF*, Hz), estimated swimming speed (*u*, ms^-1^), and body mass (*m*, kg) for saithe (*P*. *virens*) and sturgeon (*A*. *brevirostrum*). Subscript, *sd*, indicates standardized by the species-specific average, the *p* value indicates significance between predicted and observed *β* where the 95% confidence interval (CI), coefficient of determination (*r* ^2^) and sample size (*n*) are provided.
(DOCX)

S2 Table Summary of regressions between mass and tail beat frequency. Summary of regression models for predicting body mass (*m*, kg) as a function of dominant tail beat frequency (*TBF*, Hz) for saithe (*P*. *virens*) and sturgeon (*A*. *brevirostrum*) where the proportionality constant with the 95% confidence interval (CI), exponent (*β*) with standard error (SE) and 95% CI, coefficient of determination (*r* ^2^) and sample size (*n*) are provided.
(DOCX)

S3 Table Summary of regressions between tail beat frequency, maximum tail beat frequency and length. Summary of log-log regression models for predicting tail beat frequency (*TBF*, Hz) as a function of maximum tail beat frequency (*TBF*_*max*_) for saithe (*P*. *virens*) and sturgeon (*A*. *brevirostrum*), and fork length (*l*, m) a function of *TBF*_*max*_ for various fish species from [3,11], where the proportionality constant/intercept and exponent/slope (*β)* with standard errors (SE) and 95% confidence intervals (CI), coefficient of determination (*r* ^2^) and sample size (*n*) are provided.
(DOCX)
