# Supplementary material for: Scaling in Free-Swimming Fish and Implications for Measuring Size-at-Time in the Wild
Source: PLoS One. 2015 Dec 16;10(12):e0144875. doi: 10.1371/journal.pone.0144875 (PMC4684220; doi:10.1371/journal.pone.0144875)
Supplement: S1 Fig — Flow chart of the zero-crossing algorithm used to extract time-varying tail beat frequency (TBF) where Δt is the beat half period, σ is the standard deviation, E(Δt) i is the average beat half period within window i and ThS* is a tuning parameter that is a function of the range of all periods (i.e., Δt max —Δt min) in the time series multiplied by a scaling value. Finding zero-crossings is based on [11]. A typical species-specific initial window length, W for e.g., saithe is 2 seconds. (PDF) [file pone.0144875.s001.pdf]

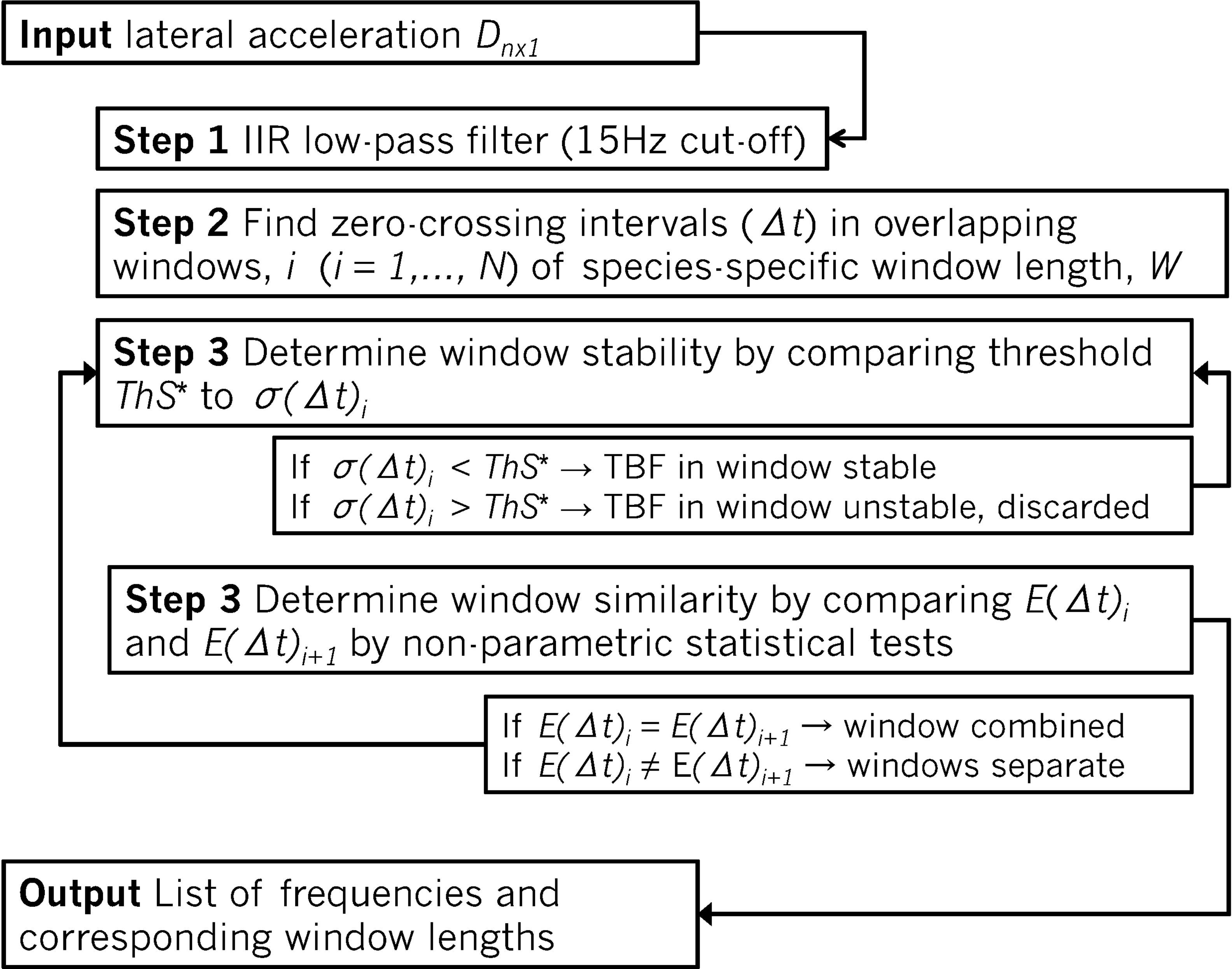

Input lateral acceleration D_{nx1}
Step 1 IIR low-pass filter (15Hz cut-off)
Step 2 Find zero-crossing intervals (Δt) in overlapping windows, i ($i = 1,\ldots, N$) of species-specific window length, W
Step 3 Determine window stability by comparing threshold ThS^* to $\sigma(\Delta t)_i$
If $\sigma(\Delta t)_i < ThS^*$ → TBF in window stable
If $\sigma(\Delta t)_i > ThS^*$ → TBF in window unstable, discarded
Step 3 Determine window similarity by comparing $E(\Delta t)_i$ and $E(\Delta t)_{i+1}$ by non-parametric statistical tests
If $E(\Delta t)_i = E(\Delta t)_{i+1}$ → window combined
If $E(\Delta t)_i \neq E(\Delta t)_{i+1}$ → windows separate
Output List of frequencies and corresponding window lengths
