# Supplementary material for: Scaling in Free-Swimming Fish and Implications for Measuring Size-at-Time in the Wild
Source: PLoS One. 2015 Dec 16;10(12):e0144875. doi: 10.1371/journal.pone.0144875 (PMC4684220; doi:10.1371/journal.pone.0144875)
Supplement: S1 File — (DOCX) [file pone.0144875.s005.docx]

**S1 File**

*Scaling Mass and TBF*

(a) *Tail beat frequency as a function of mass*

Tail beat frequency is a function of mass (S2 Fig, S1 Table) for saithe *TBF_P_* = 0.99*m^-0.29^* (*n* = 18, *r^2^* = 0.63) and for sturgeon *TBF_S_* = 2.22*m^-0.29^* (*n* = 22, *r^2^* = 0.82). The model exponents were not different (*p* = 0.99). The 95% confidence intervals (CIs) for the species-specific slopes bracket a slope of -1/3 as predicted. When beat frequencies and lengths were scaled by the species-specific average *TBF* and average mass, the best-fit model for mass (S2 Fig, S1 Table) was *TBF* = 0.90*m^-0.29^* (*n* = 40, *r^2^* = 0.63). Within species, average swimming speed was independent of *m* (*p* > 0.05, S1 Table, S3 Fig). However, when speed and mass were standardized by the species-specific averages, the relationship was marginally significant (weighted ordinary least square regression, *p* < 0.05, S1 Table, S3 Fig) and average swimming speed increased with *m* ^0.05^ (*n* = 40, S1 Table).

(b) *Mass as a function of TBF*

For each species *m* was also related to dominant *TBF* (S2 Table). For saithe, mass was proportional to *TBF ^-2.2^*, for sturgeon to *TBF ^-2.9^*. The exponents were not statistically different (*p* = 0.27) between species

*Calculation of Swimming Speed*

Since it is difficult to obtain measurements of swimming speed *in situ*, we used the species-specific prediction models from the literature to estimate swimming speed. However, different models in the literature that scale *TBF* with swimming speed appear incommensurable. For example, while using similar sized white sturgeon (*Acipenser* *transmontanus*), Long [1] predicts swimming speeds that are 3 to 4 fold lower than those provided by Cheong et al. [2]. Similarly for saithe, Videler and Hess [3] predict almost twice the swimming speed reported by Steinhausen et al. [4]. Therefore, absolute values of estimated swimming speeds, including the estimates we provide here for saithe and sturgeon, must be considered cautiously. Nevertheless, the independence of body length with swimming speed at the dominant *TBF* holds for all speed and *TBF* prediction models found in the literature. We note that for saithe, we used the Videler and Hess model because the observations corresponded well to the theoretical model proposed by Kohannim and Iwasaki [5] and Lighthill [6,7].

**Supplementary References**

1. Long JH. 1995 Morphology, mechanics, and locomotion: the relation between the notochord and swimming motions in sturgeon. *Env. Biol. Fish.* **44**, 199–211.
2. Cheong TS, Kavvas ML, Andreson EK. 2006 Evaluation of adult white sturgeon swimming capabilities and applications to fishway design. *Environ. Biol. Fish* **77(2)**, 197–208. (doi:10.1007/s10641-006-9071-y)
3. Videler JJ, Hess F. 1984 Fast continuous swimming of two pelagic predators, saithe (*Pollachius virens*) and mackerel (*Scomber scombrus*) a kinematic analysis. *J. Exp. Biol.* **109**, 209–228.
4. Steinhausen MF, Steffensen JF, Andresen NG. 2005 Tail beat frequency as a predictor of swimming speed and oxygen consumption of saithe (*Pollachius virens*) and whiting (*Merlangius merlangus*) during forced swimming. *Mar. Bio.* **148**, 197–204.
5. Kohannim S, Iwasaki T. 2014. Analytical insights into optimality and resonance in fish swimming. *J. R. Soc. Interface* **11(92)**, 20131073. (doi:10.1098/rsif.2013.1073)
6. Lighthill MJ. 1960 Note on the swimming of slender fish. *J. Fluid Mech.* **9(2)**, 305–317. (doi:10.1017/S0022112060001110)
7. Lighthill MJ. 1971 Large-amplitude elongated-body theory of fish locomotion. *Proc. R. Soc. Lond. B* **197**,125–138. (doi: 10.1098/rspb.1971.0085)
8. Hill AV. 1950 The dimensions of animals and their muscular dynamics. *Science Progr.* **38**, 209–230.
9. Sato K, Shiomi K, Watanabe Y, Watanuki Y, Takahashi A, Ponganis PJ. 2010 Scaling of swim speed and stroke frequency in geometrically similar penguins: they swim optimally to minimize cost of transport. *Proc. R. Soc. B* **277**, 707–714. (doi: 10.1098/rspb.2009.1515)
10. Kedem B. 1986 Spectral analysis and discrimination by zero-crossings. *Proc. IEEE* **74**, 1477–1493. (doi:10.1109/PROC.1986.13663)
11. Bainbridge R. 1958 The speed of swimming fish as related to size and to the frequency and amplitude of the tail beat. *J. Exp. Biol* **35**, 109–133.
