# Supplementary material for: Scaling in Free-Swimming Fish and Implications for Measuring Size-at-Time in the Wild
Source: PLoS One. 2015 Dec 16;10(12):e0144875. doi: 10.1371/journal.pone.0144875 (PMC4684220; doi:10.1371/journal.pone.0144875)
Supplement: S1 Table — Summary of allometric relations among swimming parameters based on dominant tail beat frequency (TBF, Hz), estimated swimming speed (u, ms-1), and body mass (m, kg) for saithe (P. virens) and sturgeon (A. brevirostrum). Subscript, sd, indicates standardized by the species-specific average, the p value indicates significance between predicted and observed β where the 95% confidence interval (CI), coefficient of determination (r 2) and sample size (n) are provided. (DOCX) [file pone.0144875.s006.docx]

**S1 Table** Summary of allometric relations among swimming parameters based on dominant tail beat frequency (*TBF*, Hz), estimated swimming speed (*u*, ms^-1^), and body mass (*m*, kg) in two fish species. Subscript, *sd*, indicates standardized by the species-specific average, the *p* value indicates significance between predicted and observed *β* where the 95% confidence interval (CI), coefficient of determination (*r*^2^) and sample size (*n*) are provided.

| Species | Relation | Exponent (*β*)* | 95% CI for *β* | Predicted *β* | *p* | *r*^2^ | *n* |
| --- | --- | --- | --- | --- | --- | --- | --- |
| *P. virens* | *TBF* ∝ *m^β^* | -0.29 (±0.055) | -0.41; -0.17 | -1/3^a^  -0.28^b^ | 0.40  0.94 | 0.63 | 18 |
|  | *u*^†^ ∝ *m^β^* | 0.052^†^ (±0.040) | -0.034; 0.14 | 0^a^  0.05^b^ | 0.18  0.88 | 0.11 | 18 |
| *A. brevirostrum* | *TBF* ∝ *m^β^* | -0.29 (±0.030) | -0.35; -0.22 | -1/3^a^  -0.28^b^ | 0.15  0.86 | 0.82 | 22 |
|  | *u*^‡^ ∝ *m^β^* | 0.039^‡^ (±0.029) | -0.021; 0.10 | 0^a^  0.05^b^ | 0.19  0.72 | 0.01 | 22 |
| Combined | *TBF*_sd_ ∝ m*^β^*_sd_ | -0.29 (±0.057) | -0.36; -0.22 | -1/3^a^  -0.28^b^ | 0.16  0.91 | 0.63 | 40 |
|  | *u*_sd_ ∝ m*^β^*_sd_ | 0.052^†‡^ (±0.026) | -0.001; 0.11 | 0^a^  0.05^b^ | 0.05  0.83 | 0.11 | 40 |

^a^predicted value based on [8] ^b^predicted value based on [9]
*from log-log ordinary least square slope ^†^using *u* and *TBF* model from [3] ^‡^using *u* and *TBF* model from [1]
