# Supplementary material for: Scaling in Free-Swimming Fish and Implications for Measuring Size-at-Time in the Wild
Source: PLoS One. 2015 Dec 16;10(12):e0144875. doi: 10.1371/journal.pone.0144875 (PMC4684220; doi:10.1371/journal.pone.0144875)
Supplement: S2 Table — Summary of regression models for predicting body mass (m, kg) as a function of dominant tail beat frequency (TBF, Hz) for saithe (P. virens) and sturgeon (A. brevirostrum) where the proportionality constant with the 95% confidence interval (CI), exponent (β) with standard error (SE) and 95% CI, coefficient of determination (r 2) and sample size (n) are provided. (DOCX) [file pone.0144875.s007.docx]

**S2 Table** Summary of regression models for predicting body mass (*m*, kg) as a function of dominant tail beat frequency (*TBF*, Hz) for saithe (*P. virens*) and sturgeon (*A. brevirostrum*) where the proportionality constant with the 95% confidence interval (CI), exponent (*β*) with standard error (SE) and 95% CI, coefficient of determination (*r*^2^) and sample size (*n*) are provided.

* from log-log ordinary least square intercept and slope

| Species | Relation | Proportionality  constant, *b** [95% CI] | Exponent, *β** (±SE) [95% CI] | *r*^2^ | *n* |
| --- | --- | --- | --- | --- | --- |
| *P. virens* | *m* ∝ *b* *TBF^β^* | 0.85 [0.66; 1.1] | -2.2 (±0.42) [-3.0; -1.3] | 0.63 | 18 |
| *A. brevirostrum* | *m* ∝ *b* *TBF^β^* | 13 [9.8; 16] | -2.9 (±0.31) [-3.5; -2.2] | 0.81 | 22 |
