# Supplementary material for: Scaling in Free-Swimming Fish and Implications for Measuring Size-at-Time in the Wild
Source: PLoS One. 2015 Dec 16;10(12):e0144875. doi: 10.1371/journal.pone.0144875 (PMC4684220; doi:10.1371/journal.pone.0144875)
Supplement: S3 Table — Summary of log-log regression models for predicting tail beat frequency (TBF, Hz) as a function of maximum tail beat frequency (TBF max) for saithe (P. virens) and sturgeon (A. brevirostrum), and fork length (l, m) a function of TBF max for various fish species from [3,11], where the proportionality constant/intercept and exponent/slope (β) with standard errors (SE) and 95% confidence intervals (CI), coefficient of determination (r 2) and sample size (n) are provided. (DOCX) [file pone.0144875.s008.docx]

| Species | Relation | Intercept or proportionality constant, *b** (±SE) [95% CI] | Slope or exponent, *β**  (±SE) [95% CI] | *r*^2^ | *n* |
| --- | --- | --- | --- | --- | --- |
| *P. virens* | *TBF* ∝ *b +* *β TBF_max_* | 3.9 (±0.38) [3.0; 4.7] | 2.6 (±0.32) [1.9; 3.3] | 0.79 | 18 |
| *A. brevirostrum* | *TBF* ∝ *b + β TBF_max_* | 2.6 (±0.26) [2.1; 3.2] | 1.5 (±0.18) [1.1; 1.9] | 0.78 | 22 |
| Various species^†^ | *l* ∝ *b TBF ^β^_max_* | 4.18 (±0.21) [2.76; 6.36] | -0.51 (± 0.09) [-0.71; -0.32] | 0.41 | 44 |

**S3 Table** Summary of log-log regression models for predicting tail beat frequency (*TBF*, Hz) as a function of maximum tail beat frequency (*TBF_max_*) for saithe (*P. virens*) and sturgeon (*A. brevirostrum*), and fork length (*l*, m) a function of *TBF_max_* for various fish species* from [3,11], where the proportionality constant/intercept and exponent/slope (*β****)*** with standard errors (SE) and 95% confidence intervals (CI), coefficient of determination (*r*^2^) and sample size (*n*) are provided.

^†^data from [3, 11]
* from log-log ordinary least square intercept and slope
